# Resonator-enhanced distributed Bragg reflector lasers

**DOI:** 10.1038/s41377-026-02249-x

**Published:** 2026-03-03

**Authors:** Di Yu, Zhaoting Geng, Yuhao Huang, Yitian Tong, Yu Xia, Mingfei Liu, Yaoran Huang, Chao Xiang

**Affiliations:** Department of Electrical and Electronic Engineering, The University of Hong Kong, Hong Kong, China

**Keywords:** Semiconductor lasers, Silicon photonics

## Abstract

Narrow-linewidth lasers are pivotal components for advanced optical communications, precision metrology, and microwave photonics. The drive towards low-cost, high-volume manufacturing has fueled intense interest in integrated platforms for high-coherence optical sources. However, state-of-the-art integrated low-noise lasers are fundamentally constrained by trade-offs between linewidth, tunability, and operational robustness, often sacrificing one to achieve the others. Here, we introduce and experimentally demonstrate a new class of integrated lasers—resonator-enhanced distributed Bragg reflector (RE-DBR) lasers—that overcome these limitations, simultaneously achieving ultra-narrow linewidths, wide mode-hop-free tunability, and universal turnkey operation. The RE-DBR laser architecture incorporates a grating-assisted ring resonator that serves as a compact external cavity, providing narrow-band optical feedback to enable single-wavelength lasing. Importantly, the resonator enhancement enables RE-DBR lasers to circumvent the traditional linewidth-tunability trade-off inherent to conventional DBR lasers. Furthermore, unlike self-injection-locked lasers, RE-DBR lasers maintain high optical coherence and stable operation across a broad current tuning range, enabling robust, turnkey performance. As a proof of concept, we demonstrate a RE-DBR laser with a 24 Hz Lorentzian linewidth, 34 GHz mode-hop-free tuning range, and universal turnkey operability, all realized with an external cavity of only 0.56 million loaded *Q* and a sub-4 mm^2^ footprint. These results establish RE-DBR lasers as a cost-effective, high-performance integrated alternative to bulky, expensive benchtop lasers, enabling advancement in a wide variety of applications including telecommunications, sensing, and metrology.

## Introduction

High-performance lasers are fundamental building blocks in modern optical systems, underpinning a broad range of applications such as coherent optical communications^1,2^, high-resolution spectroscopy^3^, microwave generation^4–6^, and LiDAR^7,8^. For these applications, key laser performance metrics include narrow linewidth, wide tunability, and robust operational stability. While conventional benchtop lasers can meet these requirements, integrated semiconductor lasers are increasingly favored due to their scalability, compactness, and cost-effectiveness.

Recent years have witnessed significant advances in integrated semiconductor laser technologies, particularly in terms of noise reduction^9–14^ and wavelength tunability^15–17^. Central to these improvements is the integration of narrow-band and tunable photonic reflectors into laser cavities, which both suppress noise from broadband gain media and define the lasing wavelength. Two principal reflector architectures have emerged for high-performance integrated lasers: Bragg gratings and ring resonators. Distributed Bragg reflector (DBR) lasers utilize Bragg gratings to provide wavelength-selective feedback to the gain medium, thereby enabling single-frequency operation^18^. Alternatively, self-injection-locked (SIL) lasers achieve frequency stabilization by locking the emission of a diode laser to a high-*Q* resonator via passive optical feedback, resulting in substantial linewidth narrowing^19–22^.

Advancements in low-loss photonic platforms have enabled DBR lasers to reach sub-kilohertz Lorentzian linewidths by employing ultra-low coupling coefficient ($\kappa$) gratings, typically implemented on separate low-loss photonic chips—yielding so-called extended (E-)DBR lasers^18^. These architectures also permit mode-hop-free wavelength tuning across gigahertz ranges via thermo-optic^23^, piezoelectric^24^, and electro-optic^15,25^ mechanisms, while maintaining robust operation without stringent control of injection current or ambient temperature^25,26^. However, further linewidth reduction in E-DBR lasers typically necessitates longer gratings, thereby compromising device compactness and increasing power consumption for wavelength tuning. This introduces a fundamental trade-off among linewidth, integration density, and tuning efficiency.

Self-injection-locked lasers, on the other hand, can achieve even narrower Lorentzian linewidths within a compact footprint. By leveraging ultra-high-*Q* integrated silicon nitride resonators, Lorentzian linewidths at the hertz level have been demonstrated^19,21^. Despite their impressive spectral purity, SIL lasers are highly sensitive to operational conditions^27^. Achieving optimal linewidth requires precise adjustment of the injection current to align the laser operation frequency with a specific cavity resonance. This process results in increased operational complexity, sensitivity to environmental fluctuations, and restricted mode-hop-free tunability.

Simultaneously attaining narrow linewidth, broad mode-hop-free tunability, and robust, turnkey operation is an important step for translating integrated narrow-linewidth lasers from laboratory demonstrations to practical, field-deployable systems for communications and sensing. However, the inherent architectural trade-offs in E-DBR and SIL lasers present a significant challenge: efforts to narrow the linewidth often come at the expense of wavelength tunability or operational robustness, thereby impeding the realization of fully integrated, high-performance laser sources.

In this work, we address this challenge by introducing the resonator-enhanced distributed Bragg reflector (RE-DBR) laser—a novel architecture that unites narrow linewidth, wide mode-hop-free tunability, and turnkey operation within a compact, integrated platform. The RE-DBR laser employs an on-chip grating-assisted ring resonator as an external cavity, providing single-wavelength reflection that, when coupled to a gain chip, forms a fully integrated narrow-linewidth laser source. Using a semi-analytical theoretical framework, we show that RE-DBR lasers fundamentally overcome the linewidth–tunability trade-off intrinsic to conventional DBR lasers, provided that the resonator finesse exceeds $\pi /2$. We experimentally realize this concept by butt-coupling a silicon nitride (SiN) RE-DBR external cavity chip, featuring a modest resonator finesse of approximately 90, to a III–V gain chip. The resulting hybrid integrated laser delivers over 22 mW on-chip output power, a side mode suppression ratio (SMSR) of 60 dB, a Lorentzian linewidth of 24 Hz, and a 34 GHz mode-hop-free thermo-optic tuning range, all while maintaining turnkey operation. To our knowledge, this represents the first experimental demonstration of a RE-DBR laser that quantitatively surpasses the linewidth-tunability trade-off constraining conventional DBR lasers. Furthermore, the device sustains narrow-linewidth performance across current sweeps exceeding 100 mA, thereby overcoming the operational setpoint sensitivity that limits SIL lasers. Collectively, these results establish RE-DBR lasers as a cost-effective integrated solution, capable of delivering comprehensive performance that rivals state-of-the-art benchtop laser sources.

## Results

### Laser architecture

**Fig. 1:**
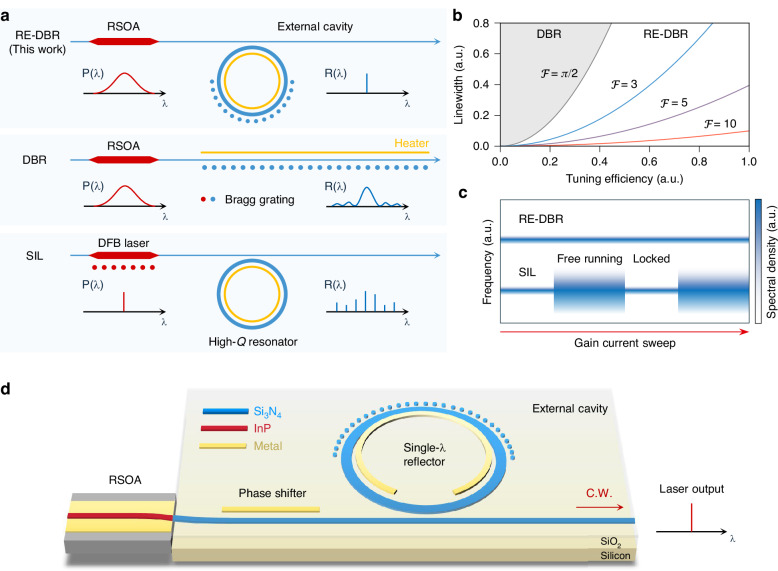
RE-DBR laser architecture and features. **a** Diagrams of the RE-DBR laser (top), a conventional DBR laser (middle), and a self-injection-locked laser (bottom). The RE-DBR laser comprises a broadband gain chip (i.e., RSOA) and a grating-assisted ring resonator that functions as a single-wavelength reflector. The DBR laser incorporates a Bragg grating that acts as the external cavity, providing narrow-band feedback to enable single-mode operation. The SIL laser comprises a DFB laser diode coupled to an external high-*Q* resonator. Intra-cavity Rayleigh scattering produces a reflection spectrum characterized by peaks at the resonant frequencies, with random amplitudes. **b** Theoretical trade-off between Lorentzian linewidth and tuning efficiency (laser wavelength shift per unit tuning power) for DBR (gray region) and RE-DBR lasers with different resonator finesse. For the RE-DBR lasers, each linewidth-tuning efficiency curve is generated by varying the effective external cavity length. The DBR region exhibits a quadratic lower bound, while the RE-DBR region surpasses this bound for resonator finesse exceeding $\pi /2$, enabling narrower linewidths and/or higher tuning efficiency. **c** Relationship between gain current and laser linewidth for RE-DBR and SIL lasers. SIL lasers achieve narrow linewidth within a limited gain current range wherein the DFB wavelength is in the proximity of a resonance of the external resonator, requiring precise bias control. In contrast, RE-DBR lasers sustain narrow linewidths across a broad range of gain currents, enabling turnkey operation. **d** Schematic of the RE-DBR laser implementation. An InP RSOA is butt-coupled to a SiN ring resonator that is partially covered by a Bragg grating, forming a hybrid integrated C-band laser. Microheaters on both the ring resonator and bus waveguide enable thermo-optic tuning

The RE-DBR laser consists of a reflective semiconductor optical amplifier (RSOA) coupled to an external cavity incorporating a grating-assisted ring resonator, as illustrated in Fig. 1a (top). When the Bragg wavelength of the grating coincides with a resonant mode of the ring resonator, the combined structure exhibits a narrow-band reflection spectrum, thereby enabling single-mode operation and low-noise lasing. Previous theoretical modeling and experimental demonstrations of grating-assisted ring resonators have established their ability to generate single-wavelength reflection spectra^28–30^. Building on these foundations, we examine the performance benefits that arise when such a single-wavelength reflector is integrated into an external-cavity laser to form the RE-DBR architecture. Specifically, we provide a systematic comparison between the RE-DBR laser and two widely adopted narrow-linewidth laser architectures—the E-DBR lasers and the self-injection-locked lasers—highlighting the distinct trade-offs these platforms exhibit among laser linewidth, tunability, and operational robustness.

In a hybrid integrated implementation, the (E-)DBR laser comprises an RSOA gain chip and an external cavity that incorporates a Bragg grating (Fig. 1a, middle). The Bragg grating provides frequency-selective feedback, enabling lasing at the grating’s Bragg wavelength. This architecture offers several advantages, including structural simplicity, single-mode operation, narrow linewidth^18,24^, and turnkey functionality^25^, establishing it as a fundamental laser design that has been extensively studied. However, an intrinsic trade-off exists in the performance of DBR lasers: it is not possible to simultaneously achieve both narrow linewidth and high tuning efficiency (defined as wavelength shift per unit tuning power). This inherent trade-off can be quantitatively expressed as (see derivation in Supplementary Section C):

1 $$\frac{\Delta \nu }{\Delta {\nu }_{0}} > 4{\left(\frac{{n}_{g,a}{L}_{a}}{{n}_{g}{\eta }_{0}}\eta \right)}^{2}$$

where $\Delta \nu$ is the Lorentzian linewidth of the DBR laser, $\Delta {\nu }_{0}$ is a reference linewidth determined by the gain chip properties and the peak reflectivity of the external cavity, ${n}_{g}$ and ${n}_{g,a}$ are the group indices for the external cavity and the gain region, respectively, ${L}_{a}$ is the length of the gain region, $\eta$ is the laser tuning efficiency, and ${\eta }_{0}$ is a parameter dependent on the material properties and waveguide geometries. The underlying physics of this linewidth-tunability trade-off is straightforward: achieving narrow linewidth requires a long Bragg grating to provide narrow-band feedback, which in turn demands higher power for tuning the material’s refractive index and, consequently, the wavelength.

The RE-DBR laser addresses this linewidth-tunability trade-off observed in conventional DBR lasers through the introduction of resonator enhancement. This enhancement effectively increases the optical path length of the grating, resulting in a narrower reflection bandwidth and higher reflectivity, surpassing the constraints imposed by the physical length of the grating. Consequently, the RE-DBR laser achieves both a lower lasing threshold and a narrower linewidth, all while maintaining a compact footprint. This enables the concurrent realization of both narrow linewidth and high tuning efficiency.

Within the framework of coupled mode theory, we derive the relationship governing the linewidth-tuning efficiency trade-off in RE-DBR lasers (see Supplementary Section C):

2 $$\frac{\Delta \nu }{\Delta {\nu }_{0}}=\frac{{\pi }^{2}}{{{\mathcal{F}}}^{2}}{\left(\frac{{n}_{g,a}{L}_{a}}{{n}_{g}{\eta }_{0}}\eta \right)}^{2}$$

Here ${\mathcal{F}}$ denotes the resonator finesse, while the remaining parameters are defined analogously to those in Eq. 1. The resonator finesse quantifies the average number of round-trips a photon undergoes before being lost through intrinsic loss or external coupling. A high finesse corresponds to a long photon lifetime, which can be achieved even in resonators with a small physical footprint. The extended photon lifetime facilitates a narrow lasing linewidth, while the compact device footprint minimizes the power required for tuning. Consequently, RE-DBR lasers incorporating high-finesse resonators enable the simultaneous realization of narrow linewidth and high tuning efficiency, as indicated by Eq. 2.

This relationship between linewidth and tuning efficiency is plotted for various values of finesse in Fig. 1b. In this figure, the x-axis represents the normalized tuning efficiency $\frac{{n}_{g,a}{L}_{a}}{{n}_{g}{\eta }_{0}}\eta$, while the y-axis corresponds to the dimensionless Lorentzian linewidth $\frac{\Delta \nu }{\Delta {\nu }_{0}}$. The feasible linewidth–tuning efficiency configurations for DBR lasers (Eq. 1) are shown in gray, while those for RE-DBR lasers (Eq. 2) are depicted using a color gradient, where the color indicates the resonator finesse. As long as the finesse exceeds $\pi /2$, the RE-DBR lasers can achieve either a narrower linewidth or a higher tuning efficiency compared to conventional DBR lasers.

Another notable advantage of RE-DBR lasers is their turnkey operability, which sets them apart from self-injection-locked lasers that are sensitive to operating setpoints. Conventional SIL lasers consist of a distributed feedback (DFB) laser coupled to an external high-*Q* resonator, as illustrated in Fig. 1a (bottom). When the DFB emission is tuned close to a resonance of the external resonator, Rayleigh scattering within the resonator provides narrow-band optical feedback to the DFB laser, resulting in linewidth narrowing. While SIL allows for ultra-narrow linewidths on integrated photonic platforms, it requires precise control of operating conditions, such as temperature and gain current, to maintain this narrow linewidth performance. If, for example, the gain current drifts outside the locking range, the laser noise characteristics revert to those of the free-running DFB laser (see Fig. 1c). This instability arises from the coexistence of dual feedback paths (from the DFB cavity and the external resonator) inherent to injection locking. In contrast, RE-DBR lasers employ a single feedback mechanism and can maintain high coherence over a wide range of temperatures and gain currents. This inherent system robustness enables turnkey operation, making the RE-DBR laser a reliable solution for integrated narrow-linewidth laser sources.

### Experimental demonstration

**Fig. 2:**
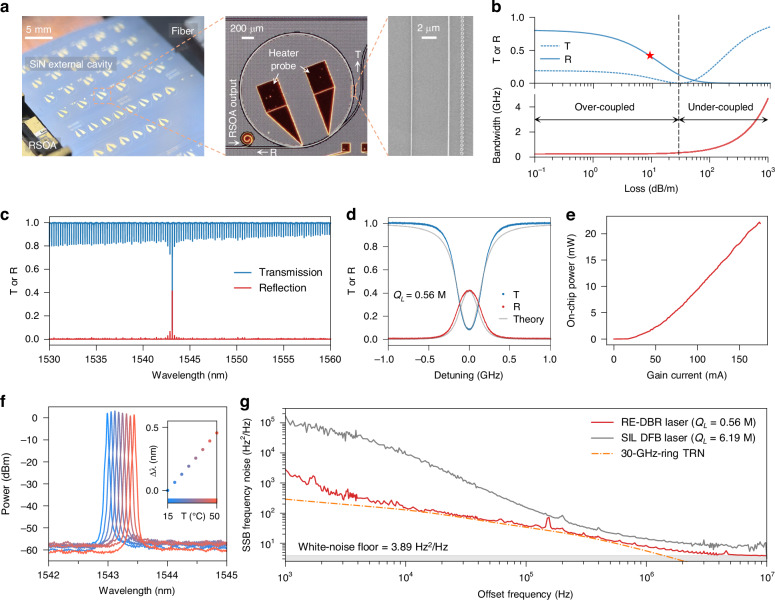
Experimental demonstration of RE-DBR laser. **a** Photograph (left), optical microscope image (middle), and scanning electron microscope (SEM) image (right) of the RE-DBR laser. **b** Semi-analytical calculations of the transmission, reflection, and bandwidth of the RE-DBR external cavity as functions of the waveguide loss. The device configuration is marked by a red star. Calculations assume a 5% resonator coupling ratio and a grating coupling strength of 0.054 cm^−1^. The strong resonator coupling ensures operation in the over-coupling regime, resulting in enhanced reflection and loss-insensitive bandwidth. **c** Measured transmission and reflection spectra of the RE-DBR external cavity, showing a dominant reflection peak near 1543 nm, corresponding to the Bragg wavelength of the grating. **d** Enlarged view of the transmission and reflection spectra around the highest reflection peak. Experimental results agree well with theoretical predictions, assuming a waveguide loss of 9 dB/m. The loaded *Q* of the resonator, extracted from the transmission resonance FWHM, is 0.56 million (M). **e** On-chip laser output power as a function of gain current. The optical power reaches 22 mW at a gain current of 174 mA. **f** Optical spectra of the RE-DBR laser at various temperatures. Lasing occurs near 1543 nm, the wavelength of maximum feedback, with a side mode suppression ratio of about 60 dB. The inset displays the laser wavelength shift as a function of temperature. Over a 35 ^◦^C temperature range, the wavelength shift is less than 0.5 nm, which indicates good thermal stability. **g** Single-sideband (SSB) frequency noise spectra of the RE-DBR laser, a SIL laser, and the thermo-refractive noise (TRN) in a SiN ring resonator with a 30 GHz free spectral range (FSR). The SIL laser uses a SiN ring resonator with a loaded *Q* of 6.19 million and a maximum reflection of about 10%. The RE-DBR laser exhibits a white-noise floor of 3.89 Hz^2^/Hz, corresponding to a Lorentzian linewidth of 24.4 Hz

Based on the design concepts of grating-assisted ring resonators, we experimentally demonstrate a hybrid integrated RE-DBR laser by butt-coupling an indium phosphide (InP)-based gain chip with a SiN RE-DBR external cavity chip (Fig. 1d). The photograph, optical microscope image, and scanning electron microscope (SEM) image of the device are presented in Fig. 2a. The gain chip employed is a commercially available, semi-butterfly-packaged C-band RSOA^31^. The RE-DBR structures are fabricated on a 100 nm-thick SiN platform, featuring an 8 *µ*m-thick buried oxide layer and a 2 *µ*m-thick oxide cladding (see Methods). Each RE-DBR structure incorporates a 2.8 *µ*m-wide bus waveguide and a 4.6 *µ*m-wide ring resonator. This ring resonator has a radius of 1 mm, yielding a free spectral range (FSR) of 30 GHz. An array of grating posts, with a period of 522 nm and a diameter of 260 nm, is positioned adjacent to the ring resonator, separated by a 1.4 *µ*m gap, to provide wavelength-selective reflection. This grating-assisted ring resonator is evanescently coupled to the bus waveguide via an asymmetric coupler, which is engineered to match the propagation constants between the single-mode bus waveguide and the fundamental mode of the ring resonator. Coupling to higher-order transverse modes is effectively suppressed due to the propagation constant mismatch. Mode converters are integrated at both ends of the external cavity chip to ensure efficient coupling to the RSOA on one side and to a single-mode fiber on the other. Following edge polishing of the external cavity chip, a butt-coupling efficiency of over 30% to the RSOA is achieved.

To optimize the geometrical parameters of the device, we develop a coupled mode theory model for the RE- DBR and perform comprehensive parameter sweeps (see Supplementary Sections A and B). Based on these calculations, we identify an optimal configuration comprising a 5% resonator coupling ratio, a grating coupling strength of 0.054 cm^−1^, and a grating length equal to half the ring circumference. This combination achieves a desirable trade-off among high reflectivity, narrow bandwidth, and a low side-lobe reflection. The calculated passive characteristics of the RE-DBR external cavity as a function of waveguide loss are presented in Fig. 2b, with the parameters of our device marked by a red star. The results reveal distinct behaviors in the under-coupling and over-coupling regimes. In the under-coupling regime, the RE-DBR external cavity exhibits low reflectivity and broad bandwidth, both of which are suboptimal for laser feedback applications. In contrast, our device operates in the over-coupling regime due to its relatively strong resonator coupling ratio, thereby providing strong and narrow-band optical feedback.

The measured transmission and reflection spectra of the RE-DBR external cavity are presented in Fig. 2c. A pronounced reflection peak is observed at 1543 nm, corresponding to the Bragg wavelength of the grating. The side-lobe reflection is 7 dB lower than the main peak, which supports single-mode lasing operation. A zoom-in view of the primary reflection peak is shown in Fig. 2d, from which we extract a peak reflectivity of 42% and a minimum transmission of 9%. These experimental results are in good agreement with theoretical calculations, assuming a waveguide loss of 9 dB/m. This level of loss is relatively high for the thin SiN platform and may be attributed to additional loss mechanisms introduced by the grating, such as out-of-plane scattering and leakage into higher-order modes. Furthermore, the resonance exhibits a loaded *Q* of 0.56 million, corresponding to a resonator finesse of 90.7, as determined from the full width at half maximum (FWHM) of the transmission dip. It is noteworthy that no mode splitting is observed due to the strong coupling between the resonator and the bus waveguide, despite the grating-induced coupling between counter-propagating modes.

We characterized the on-chip optical power of the RE- DBR laser as a function of gain current, and the results are shown in Fig. 2e. The device achieves a maximum output power of 22 mW at a gain current of 174 mA, corresponding to a slope efficiency of 0.17 mW/mA. Further improvements in laser power and slope efficiency are feasible through enhanced butt-coupling efficiency and reduced waveguide loss. The optical spectra acquired at various operating temperatures are shown in Fig. 2f. Across the temperature range of 15 ^◦^C to 50 ^◦^C, the laser consistently exhibits single-mode operation with a SMSR of 60 dB. The observed decrease in output power at elevated temperatures is attributed to thermal rollover. Furthermore, the wavelength drift remains below 0.5 nm throughout this temperature change, as shown in the inset. These results demonstrate that the RE-DBR laser has excellent thermal stability, which is at least one order of magnitude higher than that of typical III-V or III-V/silicon lasers^10^.

The single-sideband frequency noise spectra for the RE-DBR laser, a SIL DFB laser, and the thermo-refractive noise (TRN) in a SiN ring resonator with a 30 GHz FSR are shown in Fig. 2g. Frequency noise measurements were performed using the correlated self-heterodyne technique described in ref. ^32^ (see Methods). Calibration with a commercial low-noise fiber laser confirmed a measurement background noise below 0.13 Hz^2^/Hz, enabling accurate laser linewidth characterization down to the hertz level. For the SIL DFB laser, a SiN ring resonator was employed as the external cavity, with the gain current of the DFB laser finely adjusted to match a resonant mode at 1550 nm. This mode exhibits a loaded Q of 6.19 million and a peak reflection of approximately 10% (see Supplementary Section H). The occurrence of injection locking was confirmed by observing a reduction in the linewidth of the delayed self-heterodyne beatnote, measured using an electrical spectrum analyzer. The TRN of the SiN resonator was calculated based on the fluctuation-dissipation theorem and finite-element modeling (see Supplementary Section G). The RE-DBR laser approaches the TRN limit at intermediate offset frequencies between 10 kHz and 500 kHz. At higher offset frequencies (>5 MHz), the noise spectrum reaches a white noise floor of 3.89 Hz^2^/Hz, corresponding to a Lorentzian linewidth of 24.4 Hz.

Notably, although the external cavity used for SIL exhibits a loaded *Q* that is an order of magnitude higher than that of the RE-DBR external cavity, the latter nevertheless yields a narrower laser linewidth. This counter-intuitive result can be ascribed to two primary factors. First, the RE-DBR external cavity provides stronger reflection compared to the ring resonator utilized in the SIL configuration. Second, the linewidths of the SIL and RE-DBR lasers exhibit different dependencies on the external resonator reflection, $R$. Specifically, the SIL laser linewidth scales as ${R}^{-1}$, whereas the RE-DBR laser linewidth is proportional to ${\left(\mathrm{ln}R\right)}^{2}$ (see Supplementary Section D for details). In the regime of low external feedback—relevant to our case due to butt-coupling losses—the RE-DBR laser can thus achieve a narrower linewidth than the SIL laser. Collectively, RE-DBR’s stronger feedback and ${\left(\mathrm{ln}R\right)}^{2}$ linewidth dependence outperform SIL’s ${R}^{-1}$ scaling at low reflectivity. As a result, the RE-DBR laser attains a narrow Lorentzian linewidth without necessitating a high-*Q* resonator, thereby relaxing fabrication constraints.

### Mode-hop-free tunability

Simultaneously achieving both a narrow linewidth and a large mode-hop-free tuning range poses significant challenges for conventional laser architectures, such as DFB lasers and DBR lasers. In these systems, a narrow linewidth is associated with a reduced longitudinal mode FSR, which inherently limits the mode-hop-free tuning range^17^. Here, we experimentally demonstrate that the RE-DBR laser can overcome this limitation by achieving mode-hop-free tuning over a range of 34 GHz, which is equivalent to 33 times the longitudinal mode FSR.

**Fig. 3:**
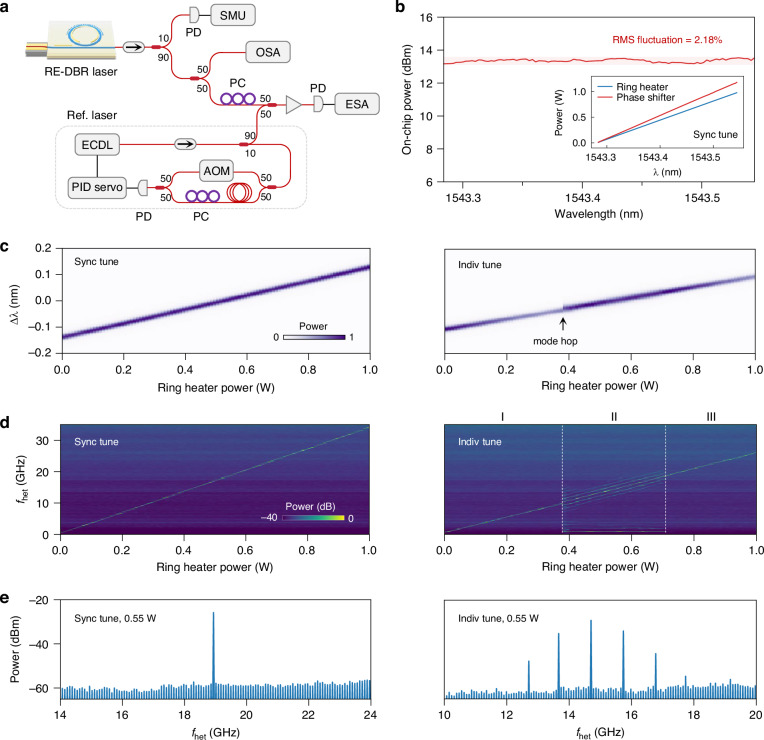
Demonstration of mode-hop-free tunability. **a** Schematic of the experimental setup. The laser output is split into three paths for power measurement, optical spectrum analysis, and heterodyne detection. The reference source for heterodyne detection is an external cavity diode laser (ECDL) stabilized by a frequency-locking loop. Photodetectors (PDs), polarization controllers (PCs), source measure unit (SMU), optical spectrum analyzer (OSA), acousto-optic modulator (AOM), and electrical spectrum analyzer (ESA) are also used in the experiment. **b** Laser output power as a function of wavelength during synchronous tuning. The root-mean-square (RMS) fluctuation in laser power across the tuning process is 2.18%. The inset shows the heater power applied during the synchronous tuning. **c** Optical spectra under synchronous tuning (left) and individual ring heater tuning (right); the discontinuity in the spectrum indicates a mode hop. **d** Electrical spectra of the heterodyne beatnote under synchronous tuning (left) and individual tuning (right). Synchronous tuning yields a beatnote frequency that drifts continuously and linearly with ring heater power, demonstrating mode-hop-free tuning over 34 GHz; individual tuning leads to abrupt beatnote changes, validating the occurrence of mode hops. **e** Beatnote frequency spectra at 0.55 W ring heater power for synchronous (left) and individual (right) tuning. Synchronous tuning corresponds to a single-peak spectrum (single-mode lasing), while individual tuning results in a multi-peak profile (multi-mode lasing). From the frequency spacing of these peaks, the longitudinal mode FSR is determined to be 1.02 GHz

To verify the absence of mode hops during tuning, we implemented the experimental setup shown in Fig. 3a. The laser output is coupled into a single-mode fiber and divided into three channels for power monitoring, optical spectrum analysis, and heterodyne detection. Mode-hop-free operation is confirmed by observing stable output power, continuous tuning of the laser wavelength, and uninterrupted shifts in the heterodyne beatnote frequency. Heterodyne detection is performed using an external cavity diode laser (ECDL), frequency-stabilized via a feedback loop (see Supplementary Section I). The resulting beatnote is analyzed using an electrical spectrum analyzer (ESA) with a resolution bandwidth of 1 MHz—significantly narrower than the 2.5 GHz frequency resolution of the optical spectrum analyzer (OSA). This setup enables clear identification of single-mode operation, multi-mode lasing, and the presence or absence of mode hops.

Our thermo-optic tuning mechanism utilizes two independently controlled heaters: a phase shifter located adjacent to the bus waveguide, and a ring-shaped heater positioned above the RE-DBR (Fig. 1d). The ring heater is used to tune the reflection wavelength of the resonator, while the phase shifter compensates for any mismatch between the longitudinal cavity mode and the resonator reflection wavelength, ensuring that the laser operates at the wavelength of peak reflection to enhance power stability. By adjusting the heater powers synchronously such that the longitudinal mode and the reflection wave-length shift at identical rates, mode-hop-free tuning can be attained.

During synchronous tuning, the applied power was proportionally allocated between the ring heater and phase shifter, with the proportionality factor optimized to minimize fluctuations in the laser output power throughout the tuning range. This method achieved a root-mean-square (RMS) power variation of only 2.18% during wavelength tuning over a range of 0.26 nm, as shown in Fig. 3b, demonstrating excellent power stability. During this process, the ring heater power was varied from 0 to 1 W, while the phase shifter power was swept from 0 to 1.2 W, as depicted in the inset. The corresponding optical spectrum and heterodyne beatnote spectrum under synchronous tuning are shown in Fig. 3c (left) and Fig. 3d (left), respectively. Both spectra exhibit single-peak profiles, confirming single-mode laser operation. Furthermore, the laser wavelength and beatnote frequency display continuous and linear tuning with respect to the ring heater power, without any observable discontinuities. These results demonstrate mode-hop-free tuning over a range of 34 GHz—a value limited only by the material’s thermo-optic coefficient, which could be extended by migrating our design to silicon-on-insulator platforms.

For comparison, we investigated the behavior of the RE-DBR laser under individual tuning of the ring heater, with no power applied to the phase shifter, using the same power sweep range as in the synchronous tuning experiment. The optical and heterodyne beatnote spectra obtained under individual tuning are presented in Fig. 3c (right) and Fig. 3d (right). A discontinuity in the optical spectrum is observed at a ring heater power of 0.38 W, coinciding with a transition in the beatnote from a single-peak (regime I) to a multi-peak profile (regime II). This indicates the onset of a mode hop and a transition from single-mode to multi-mode laser operation. As the ring heater power is further increased to 0.71 W, the heterodyne beatnote reverts to a single-peak profile (regime III), indicating a return to single-mode operation.

Specifically, the beatnote spectra at a ring heater power of 0.55 W are shown in Fig. 3e. In the case of individual tuning, the beatnote exhibits multiple, equally spaced frequency components. The measured spectral separation of 1.02 GHz corresponds to an effective cavity length of approximately 10 cm, which is significantly longer than the length of the grating (3 mm). This quantitatively demonstrates the extent of resonator enhancement achieved in the device, resulting in an extended cavity length and a dramatic reduction in the laser linewidth.

### Turnkey operation

The ultra-narrow linewidth observed in the RE-DBR laser is primarily ascribed to the narrow reflection bandwidth and high reflectivity of its external cavity. These attributes stem from the precise alignment between the grating’s Bragg wavelength and a resonant mode of the ring resonator. Importantly, this alignment is determined by the material composition and structural design of the external cavity, independent of any active tuning mechanisms. Consequently, the narrow-linewidth characteristic of the RE-DBR laser is expected to be intrinsically robust against variations in operating conditions and readily achievable via turnkey operation.

**Fig. 4:**
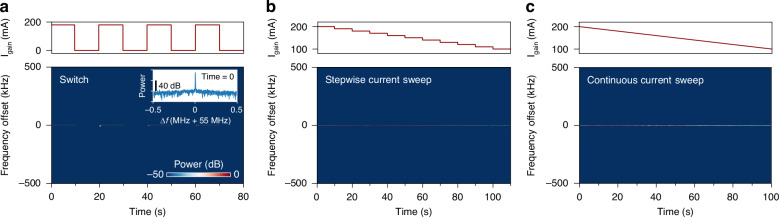
Turnkey narrow-linewidth operation. **a** Electrical spectrum of the delayed self-heterodyne beatnote under square-wave current modulation, alternating between 0 mA (off) and 180 mA (on). The inset displays the beatnote spectrum at the initial time. Each time the laser is switched on, the beatnote exhibits a consistently narrow linewidth, indicating immediate recovery of high coherence. **b**, **c** Beatnote spectra measured during a stepwise current sweep (**b**) and a continuous current sweep (**c**) from 200 mA to 100 mA. In both cases, the linewidth remains narrow across the entire current range, demonstrating the robustness of the laser coherence to changes in the current setpoint

In the following, we experimentally demonstrate turnkey, narrow-linewidth operation of the RE-DBR laser. Specifically, we validate the narrow-linewidth characteristic under both laser switching operations and a range of injection current sweeps to demonstrate robust and universal turnkey operability. To emulate laser switching and evaluate linewidth stability under different injection currents, we applied current modulation to the device. The laser’s noise characteristics were assessed using the delayed self-heterodyne technique. A relatively short optical delay line was used to suppress the effects of long-term wavelength drift, allowing us to isolate and quantify the laser’s short-term frequency stability (see Methods). Figure 4a (inset) shows the electrical spectrum of the heterodyne beatnote at a gain current of 180 mA. The spectrum features a single frequency component at 55 MHz, corresponding to the first-order frequency shift introduced by the AOM, with an effective linewidth on the order of kHz, indicating high coherence in the laser output.

To further assess the performance of the RE-DBR laser, we implemented three current modulation schemes: square-wave, stepwise-sweep, and linear-sweep modulation, as depicted in the upper panels of Fig. 4a–c, respectively. For square-wave modulation, the gain current alternated between a high level (180 mA, “on”) and zero (0 mA, “off”). The corresponding beatnote spectra, shown in Fig. 4a (lower), consistently exhibit a narrow linewidth whenever the gain current is at the high level. This test was repeated multiple times with similar results, confirming both the feasibility and the repeatability of turnkey narrow-linewidth operation.

In the stepwise-sweep and linear-sweep experiments, the gain current was varied from 200 mA to 100 mA. The resulting beatnote spectra are presented in Fig. 4b (lower) and Fig. 4c (lower), respectively. In both current sweeps, the beatnote consistently maintains a narrow linewidth and its power varies smoothly, indicating mode-hop-free, narrow-linewidth operation across a wide range of current settings. Collectively, these results confirm the operational stability and universal turnkey nature of the RE-DBR laser’s narrow-linewidth emission.

## Discussion

The turnkey, tunable, and narrow-linewidth integrated laser sources developed in this work have broad applicability in fields such as precision metrology, remote sensing, and microwave photonics. For example, in precision metrology, the lasers’ low-frequency noise enables frequency comb generation for high-resolution dual-comb spectroscopy^33–35^. In remote sensing applications, their tunability and narrow linewidth enable LiDAR systems to achieve high ranging accuracy^7,15,36^. In microwave photonics, these integrated lasers deliver low-jitter optical carriers. Such carriers are crucial for producing high-spectral-purity microwave signals through optical frequency division^4–6,37,38^ and optoelectronic oscillators^39–41^.

To better meet the demands of these practical applications, further improvements in modulation bandwidth and tuning range are desirable. In the present demonstration, both metrics are constrained by thermo-optic tuning and by the relatively small thermo-optic coefficient of silicon nitride. These limitations, however, are not intrinsic to the RE-DBR architecture and can be overcome by implementing the design in alternative material platforms. For instance, silicon offers a large thermo-optic coefficient for an expanded thermo-optic tuning range^42^, while lithium niobate exhibits a strong Pockels effect, enabling high-speed electro-optic modulation^8,25^.

Fabrication robustness is another key factor for large-scale deployment. The RE-DBR laser shows strong tolerance to fabrication variations: among 36 devices tested in the most recent iteration—spanning nominal grating coupling strengths from 0.02 cm^−1^ to 0.1 cm^−1^ and resonator coupling ratios from 2% to 25%—14 devices (yield 39%) exhibited single-peak reflection spectra compatible with stable single-mode lasing. The primary yield limitation is the random mismatch between the grating Bragg wavelength and a ring resonance. We anticipate that yield can be significantly enhanced in a production-oriented design without extensive parameter sweeps, thereby enabling low-cost, high-volume manufacturing.

Several promising directions remain for future work. First, by reducing the resonator coupling strength, it is possible to realize a RE-DBR laser with a Lorentzian linewidth at the hertz level, requiring only a loaded *Q* of several million—a readily achievable target with current SiN platforms. Second, the level of integration may be further advanced through the use of advanced fabrication techniques, including heterogeneous integration^43^ and micro-transfer printing^44^, which can improve system stability and pave the way towards mass production and commercial deployment.

## Materials and methods

### Coupled mode theory for RE-DBR

We employed a semi-analytical model to describe the external cavity of the RE-DBR system based on coupled mode theory^45^. The detailed derivation is provided in Supplementary Section A; here, we provide a concise summary of the key results.

Consider a ring resonator characterized by a circumference ${l}_{r}$, an intensity loss coefficient $\rho$, and a propagation constant $\beta$. The round-trip amplitude transmission coefficient is given by: ${t}_{0}=\exp \left(-\rho {l}_{r}/2-j\beta {l}_{r}\right)$. The ring resonator is evanescently coupled to a bus waveguide, with a self-coupling coefficient ${\tau }_{1}$ and a cross-coupling coefficient ${\tau }_{2}$. To implement the RE-DBR, a Bragg grating partially covers the resonator, introducing wavelength-selective reflection. The grating’s single-trip amplitude transmission and reflection coefficients are denoted as ${t}_{g}$ and ${r}_{g}$, respectively. Using these parameters, the amplitude transmission and reflection coefficients of the composite RE-DBR feedback structure, as well as the effective cavity length, can be expressed as follows:

3 $$\begin{array}{c}t={\tau }_{1}+{\tau }_{2}{\tau }_{2}^{* }{t}_{0}\,\frac{{\tau }_{1}^{* }{t}_{0}-\left|{t}_{g}\right|}{1-2{\tau }_{1}^{* }\left|{t}_{g}\right|{t}_{0}+{\left({\tau }_{1}^{* }\right)}^{2}{t}_{0}^{2}}\\ r=j\frac{{\tau }_{2}{\tau }_{2}^{* }\left|{r}_{g}\right|{t}_{0}}{1-2{\tau }_{1}^{* }\left|{t}_{g}\right|{t}_{0}+{\left({\tau }_{1}^{* }\right)}^{2}{t}_{0}^{2}}\\ {L}_{{\rm{eff}}}=\frac{1-{\left({\tau }_{1}^{* }{t}_{0}\right)}^{2}}{2\left[1-2{\tau }_{1}^{* }\left|{t}_{g}\right|{t}_{0}+{\left({\tau }_{1}^{* }{t}_{0}\right)}^{2}\right]}{l}_{r}\end{array}$$

The relevant parameters (${\tau }_{1}$, ${\tau }_{2}$, $\beta$, ${t}_{g}$, and ${r}_{g}$) are obtained through numerical simulations, following the methods described in refs. ^46^^,^^47^.

### Device fabrication

The SiN RE-DBR chip is fabricated at the HKUST Nanosystem Fabrication Facility, following the process described in ref. ^19^. The fabrication begins with a commercial 6-inch-diameter silicon nitride (SiN) wafer comprising a 100 nm-thick SiN cladding layer atop an 8 *µ*m-thick buried oxide. The RE-DBR structures are defined in the SiN layer via 248 nm deep ultraviolet (DUV) lithography followed by reactive ion etching (RIE). Subsequently, a 2 *µ*m-thick oxide cladding is deposited using plasma-enhanced chemical vapor deposition (PECVD), followed by high-temperature annealing to reduce hydrogen content and minimize waveguide loss. Titanium/platinum (Ti/Pt) heaters and probe metals are then deposited and patterned using a standard lift-off process.

### Laser linewidth measurement

The laser noise spectra are characterized using the correlated self-heterodyne technique^32^. The experimental setup comprises a delayed self-heterodyne module, two balanced photodetectors (BPDs, Thorlabs PDB415C-AC), and an oscilloscope (Siglent SDS7304A). The delayed self-heterodyne module consists of a 1 km-long fiber delay line, a 55 MHz AOM (Brimrose AMM-55-8-70-2FP), and a polarization controller. The optical output from the self-heterodyne module is split into four channels and detected by the two BPDs, resulting in two beatnote signals. These beatnote signals are simultaneously recorded by the oscilloscope, and their cross-correlation is computed to extract the spectral density of common-mode frequency noise. To correct for the Mach-Zehnder interferometer filtering effect inherent to the self-heterodyne module, a gain factor is applied to the cross-correlation result, yielding the frequency noise spectrum of the laser. To minimize technical noise in the measurements, the lasers under test are powered by a low-noise current source (Newport LDX-3620B) with a RMS current noise below 70 nA. Furthermore, the operating temperature is stabilized at 20.00 °C with fluctuations under 10 mK using a thermoelectric cooler controlled by a Thorlabs TC300B.

### Delayed self-heterodyne measurement

The standard delayed self-heterodyne technique was employed to characterize the laser noise properties under current modulation. The experimental setup comprises a fiber-based Mach-Zehnder interferometer, in which one arm incorporates a 40-meter-long fiber delay line, while the other arm contains a polarization controller and a 55 MHz AOM. The outputs from the two arms are recombined, and the resulting interference signal is detected by a BPD, generating an electrical beatnote that encodes the coherence properties of the laser output. The beatnote is subsequently analyzed using an electrical spectrum analyzer (ESA, Keysight N9040B) to acquire its frequency spectra, as shown in Fig. 4.

### Performance comparison of integrated narrow-linewidth laser sources

We benchmark the performance of our RE-DBR laser against state-of-the-art integrated narrow-linewidth laser sources by comparing several key figures of merit. These include the Lorentzian linewidth, mode-hop-free tuning range, power stability during wavelength tuning, maximum output power, tuning efficiency, and device footprint. As summarized in Table 1, prior integrated lasers with comparable or narrower linewidths typically exhibit limited tunability and/or reduced scalability. By contrast, the RE-DBR laser is, to our knowledge, the first integrated platform that simultaneously achieves a sub-100 Hz Lorentzian linewidth and tens-of-GHz mode-hop-free tuning within a mm^2^-scale footprint.

**Table 1:** Performance comparison of integrated narrow-linewidth laser sources

Laser Architecture	Lorentzian Linewidth (Hz)	Mode-hop-free Tunability (GHz)	Power Stability (dB)	Maximum Power (mW)	Tuning Effic. (GHz/W)	Device Footprint (mm^2^)	Reference
**Si**_**3**_**N**_**4**_ **external-cavity lasers**							
RE-DBR	24	34	±0.19	22^‡^	25	3.1	**This Work**
Vernier	400	3	n/a	6	n/a	0.97	^49^
Vernier	2.2 × 10^3^	28	±0.5	24	13.75	4.3	^17^
Vernier	2.9 × 10^3^	56	±0.9	22.5	53	3.6	^50^
Vernier	2.4 × 10^3^	20	n/a	0.2	83	*<* 55	^51^
Ring SIL	1.2	n/a	n/a	< 30^‡^	n/a	35	^19^
Ring SIL	34.2	n/a	n/a	14.8	193	1.13	^52^
Ring SIL	8 × 10^3^	n/a	n/a	5^‡^	n/a	0.05	^53^
Ring SIL	13 × 10^3^	n/a	n/a	1.66	n/a	0.045	^54^
E-DBR	2.5 × 10^3^	40	*>* ±1^†^	25	140	6.5	^24^
**SiO**_**2**_ **external-cavity lasers**							
Ring SIL	27	*>* 0.66	n/a	0.83	n/a	70	^55^
**Si external-cavity lasers**							
Vernier	4.7 × 10^3^	225	n/a	0.7	375	3.4	^16^
Vernier	5.7 × 10^3^	375	±0.4	3.8^‡^	300	3.2	^42^
Vernier	2 × 10^3^	31	n/a	11	625	1.7	^56^
**LiNbO**_**3**_ **external-cavity lasers**							
E-DBR	167	24	n/a	4.1	n/a	10	^15^
E-DBR	2.7 × 10^3^	10	n/a	17	n/a	7.25	^25^
**Hybrid Si**_**3**_**N**_**4**_**/LiNbO**_**3**_ **external-cavity lasers**							
Ring SIL	3 × 10^3^	1.2	n/a	0.15	n/a	0.58	^8^

^†^Estimated from optical spectrum data. ^‡^On-chip output power is shown whereas other values represent fiber-coupled power. Power stability is reported as peak-to-peak fluctuation. The Bragg grating footprint is estimated as the product of a 1 mm width and the device length. *E-DBR* extended distributed Bragg reflector, *SIL* self-injection locked, *n/a* not available

## Supplementary information

Supplementary Information for: Resonator-enhanced distributed Bragg reflector lasers

## Data Availability

The data and code used to produce the plots within this work are available at 10.5281/zenodo.15875618^48^.
