# Supplementary material for: Resonator-enhanced distributed Bragg reflector lasers
Source: Light Sci Appl. 2026 Mar 3;15:142. doi: 10.1038/s41377-026-02249-x (PMC12953789; doi:10.1038/s41377-026-02249-x)
Supplement: Supplementary file 1 — Supplementary Information for: Resonator-enhanced distributed Bragg reflector lasers [file 41377_2026_2249_MOESM1_ESM.pdf]

Supplementary Information for: Resonator-enhanced distributed Bragg reflector lasers

Di Yu[1,*], Zhaoting Geng[1,*], Yuhao Huang[1,*], Yitian Tong[1], Yu Xia[1], Mingfei Liu[1], Yaoran Huang[1], Chao Xiang[1,†]
[1]Department of Electrical and Electronic Engineering, The University of Hong Kong, Hong Kong, China
*These authors contributed equally to this work.
†Corresponding author: cxiang@eee.hku.hk

CONTENTS

A. Coupled mode theory for RE-DBR 2

B. Parameter optimization of laser external cavity 3

C. Trade-off between linewidth and tuning efficiency 4
1. DBR laser 4
2. RE-DBR laser 5

D. Noise characteristics of self-injection-locked and RE-DBR lasers 5
1. SIL laser 5
2. RE-DBR laser 6

E. Fabrication process of external cavity chip 7

F. Geometry optimization of edge coupler for efficient butt-coupling to RSOA 7

G. Simulation of thermo-refractive noise in optical cavity 7
1. Fluctuation-dissipation theorem 8
2. Thermo-refractive noise in optical cavity 8
3. Simulation of thermo-refractive noise in ring resonator 9

H. Properties of the resonance mode for self-injection locking 10

I. Phase noise spectra of the reference laser for heterodyne detection 11

J. Laser frequency response under direct modulation 11

K. Thermal stability of RE-DBR 12

References 13

Appendix A: Coupled mode theory for RE-DBR

We adopt the semi-analytical method developed by Kang et al. [1, 2] to calculate the transmittance and reflectance characteristics of a resonator-enhanced distributed Bragg reflector (RE-DBR). This method is computationally efficient and facilitates the optimization of design parameters for enhanced laser performance.

Consider a RE-DBR structure as shown in Fig. S1. The setup consists of a microring resonator evanescently coupled to a bus waveguide, with a periodic array of grating posts arranged along a portion of the ring. Assuming that both the bus waveguide and the ring resonator support a single transverse mode, the local optical field can be described by the complex amplitude of this mode. The relevant optical field amplitudes are defined in Fig. S1. The transmittance and reflectance of the resonator are given by $T = |t|^2 = |B_1/A_1|^2$ and $R = |r|^2 = |A_2/A_1|^2$.

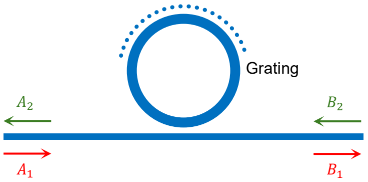

Extended Data Fig. S1: Schematic diagram of the RE-DBR. A Bragg grating partially covers the ring resonator, which is evanescently coupled to a bus waveguide.

Combining the coupled mode equations for the directional coupler, Bragg grating, and ring resonator [3–5] and the implicit condition that $B_2 = 0$ (no light returns from the output port), the amplitude transmission and reflection coefficients of the RE-DBR are found to be [6]:

$$\begin{aligned} t &= \tau_1 + \tau_2\tau_2^* t_0 \frac{\tau_1^* t_0 - |t_g|}{1 - 2\tau_1^*|t_g|t_0 + (\tau_1^*)^2 t_0^2} \\ r &= j\frac{\tau_2\tau_2^*|r_g|t_0}{1 - 2\tau_1^*|t_g|t_0 + (\tau_1^*)^2 t_0^2}, \end{aligned} \quad \text{(S1)}$$

where $t_0 = \exp(-\rho l_r/2 - j\beta_{\text{ring}} l_r)$ is the roundtrip transmission coefficient of the ring resonator, with ρ the intensity attenuation coefficient, l_r the ring circumference, and β_{ring} the propagation constant of the ring. The self-coupling coefficient and cross-coupling coefficients of the directional coupler are $\tau_1 = [\cos(ql_c) + j\frac{\delta}{q}\sin(ql_c)]e^{-j\delta l_c}$ and $\tau_2 = -j\frac{\kappa_c}{q}\sin(ql_c)e^{-j\delta l_c}$, respectively, where the effective coupling strength is $q = \sqrt{\kappa_c^2 + \delta^2}$, and l_c, κ_c, and $\delta = (\beta_{\text{ring}} - \beta_{\text{bus}})/2$ denote the length, the coupling strength and the propagation constant mismatch, respectively. Finally, t_g and r_g are the amplitude transmission and reflection coefficients of the Bragg grating, given by [3]

$$\begin{aligned} t_g &= \frac{\rho_g}{\rho_g\cos(\rho_g l_g) + j\phi\sin(\rho_g l_g)}\exp(j\phi l_g) \\ r_g &= -\frac{j\kappa_g\sin(\rho_g l_g)}{\rho_g\cos(\rho_g l_g) + j\phi\sin(\rho_g l_g)}, \end{aligned} \quad \text{(S2)}$$

where $\phi = \beta_{\text{ring}} - N\pi/\Lambda$ and $\rho_g = \sqrt{\phi^2 - \kappa_g^2}$, with N the grating order, Λ the grating period, κ_g the grating coupling coefficient, and l_g the grating length.

In the derivation, it is important to ensure global consistency in the eigenmode basis so that the continuity condition remains valid and no unphysical abrupt phase change appears in the optical field. When the propagation constants of the ring resonator and the bus waveguide are matched, Eq. S1 reduces to the formulation established in [2].

Eq. S1 provides an efficient method for calculating the transmittance and reflectance spectra of a RE-DBR. This approach eliminates the need for resource-intensive full three-dimensional numerical simulations. Instead, it only requires simulating the waveguide propagation constants and coupling strengths for the specific modes and wavelengths of interest. These simulated values can then be substituted into the equation to determine the transmission and reflection coefficients, which in turn yield the transmittance and reflectance spectra of the RE-DBR.

The effective cavity length of the RE-DBR is given by $L_{\text{eff}} = \frac{c}{2n_g}\frac{\partial\phi}{\partial\omega}$, where ϕ is the phase of the amplitude reflection coefficient $r = |r|e^{-j\phi}$, and n_g is the group index of the ring. Specifically, at the wavelength of maximum reflection,

where $\frac{\partial |r|}{\partial \omega} = 0$, it follows that $\frac{\partial r}{\partial \omega} = -j\frac{2n_g r}{c} L_{\mathrm{eff}}$. By differentiating Eq. S1 with respect to frequency, we obtain an expression for the effective cavity length at the wavelength of maximum reflection:

$$L_{\mathrm{eff}} = \frac{1 - (\tau_1^* t_0)^2}{2[1 - 2\tau_1^* |t_g| t_0 + (\tau_1^* t_0)^2]} l_r. \tag{S3}$$

Appendix B: Parameter optimization of laser external cavity

The coupled mode theory introduced in the previous section provides a computationally efficient approach for calculating the passive characteristics of the RE-DBR. This enables us to determine the optimal design parameters needed to balance transmission, reflectivity, effective cavity length, and side-lobe reflection suppression ratio to enhance the performance of the RE-DBR laser. We perform a parameter sweep over the grating coupling strength κ_g and the resonator coupling coefficient $\kappa_c l_c$; the resulting calculated passive properties of the RE-DBR are presented in Fig. S2. All calculations assume a waveguide loss of 9 dB m^{-1}. The parameter configuration used in our experimental demonstration is indicated by a white star, selected to satisfy several competing figures of merits, including high transmission, high reflection, long effective cavity length, and large side-lobe reflection suppression, thereby enabling high-power, narrow-linewidth lasers that exhibit stable single-mode operation.

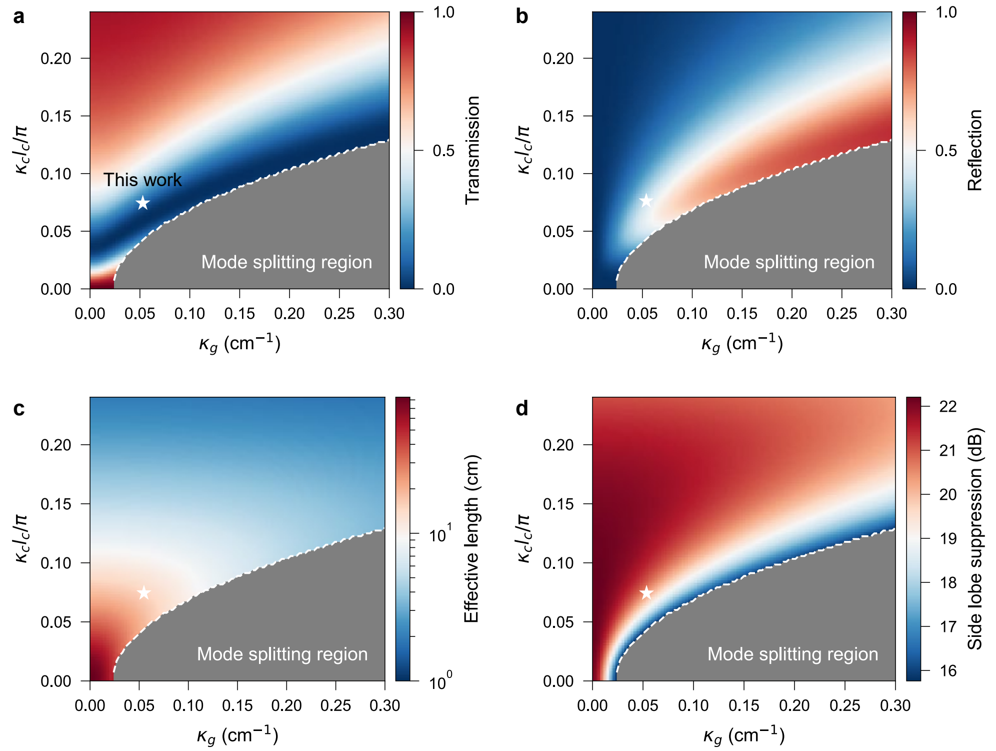

Extended Data Fig. S2: Calculated passive characteristics of the RE-DBR. **a**, Transmission at the wavelength of maximum reflection. **b**, Maximum reflection. **c**, Effective cavity length at the wavelength of maximum reflection. **d**, Side lobe reflection suppression ratio, defined as the ratio of the maximum reflection to the second highest peak reflectivity. These passive properties of the RE-DBR are calculated as functions of the grating coupling strength κ_g and the resonator coupling coefficient $\kappa_c l_c$.

Appendix C: Trade-off between linewidth and tuning efficiency

1. DBR laser

DBR lasers exploit an external cavity of Bragg grating to achieve single-mode operation and narrow linewidth, wherein the narrow-band reflection spectrum of the external cavity plays a key role. Denote the reflectivity of the front mirror (external cavity) and the back mirror by $r(\omega) = |r|e^{-j\phi}$ and r_1, respectively. The laser's Lorentzian linewidth has the following form [7, 8]:

$$\Delta\nu = \Delta\nu_0 \frac{1}{(1 + \frac{\alpha_H}{\tau_0}\frac{d\ln|r(\omega)|}{d\omega} + \frac{1}{\tau_0}\frac{d\phi}{d\omega})^2}, \tag{S4}$$

where $\Delta\nu_0$ is the Lorentzian linewidth of a Fabry-Perot (FP) diode laser with mirror reflectivities r_1 and $|r(\omega)|$; α_H is the amplitude-phase coupling coefficient (also known as the linewidth enhancement factor or Henry factor), and $\tau_0 = 2n_{g,a}L_a/c$ is photon roundtrip time in the active region, with $n_{g,a}$ and L_a being the group index and length of the active region, respectively.

If the laser operates at the wavelength of maximum reflection from the external cavity, and the effective cavity length of the external cavity L_{eff} is much larger than the length of the active region L_a, the expression for the Lorentzian linewidth simplifies to

$$\Delta\nu = \Delta\nu_0 \frac{1}{(1 + \frac{n_g L_{\text{eff}}}{n_{g,a}L_a})^2} \approx \Delta\nu_0 (\frac{n_{g,a}L_a}{n_g L_{\text{eff}}})^2, \tag{S5}$$

where n_g is the group index of the external cavity waveguide. This equation applies to general external cavity lasers, including DBR lasers and RE-DBR lasers.

Specifically, the effective cavity length of a Bragg grating is given by [8]

$$L_{\text{eff}} = \frac{\tanh(\kappa L)}{2\kappa}, \tag{S6}$$

where κ and L are the mode coupling coefficient and length of the grating, respectively. Substituting Eq. S6 into Eq. S5 and noting that $\tanh(x) < x$ for all $x > 0$, it follows that

$$\frac{\Delta\nu}{\Delta\nu_0} > 4(\frac{n_{g,a}L_a}{n_g L})^2 \tag{S7}$$

In thermo-optic or electro-optic tuning, the wavelength tuning efficiency of the grating, η, defined as wavelength shift per unit tuning power $\frac{d\lambda}{dP}$, is inversely proportional to the length of the external cavity: $\eta = \frac{\eta_0}{L}$, where η_0 is the tuning efficiency of a unit-length grating. In fact, the thermo-optic tuning efficiency of a Bragg grating is given by

$$\eta_{\text{th}} = \frac{dT}{dP}\frac{d\lambda_B}{dT} = \frac{2\Lambda R_{\text{th}}}{N}\frac{dn_{\text{eff}}}{dT}, \tag{S8}$$

where T is the temperature, P the tuning power, λ_B the Bragg wavelength, N the grating order, Λ the grating period, R_{th} the thermal resistance, and n_{eff} the effective mode index. Since the thermal resistance of the external cavity is inversely proportional to its physical length, the thermo-optic tuning efficiency η_{th} is likewise inversely proportional to the external cavity length as well. A similar scaling law applies to electro-optic tuning, where electrical impedance plays the role of thermal resistance. It is worth mentioning that deviations from this scaling law have been reported in certain tuning mechanisms, such as piezoelectrical tuning [9], in which the geometrical deformation—rather than index change—dominates the phase shift.

Substitute $\eta = \frac{\eta_0}{L}$ into Eq. S7, we obtain the linewidth-tuning efficiency trade-off for DBR lasers:

$$\frac{\Delta\nu}{\Delta\nu_0} > 4(\frac{n_{g,a}L_a}{n_g \eta_0}\eta)^2 \tag{S9}$$

2. RE-DBR laser

The RE-DBR lasers can surpass the linewidth-tuning efficiency trade-off expressed in Eq. S9 due to resonator enhancement. To clarify this, we establish the relationship between linewidth and tuning efficiency for the RE-DBR lasers. By combining Eq. S3 and Eq. S5, we obtain an expression for the Lorentzian linewidth of RE-DBR lasers:

$$\Delta\nu = \Delta\nu_0 \left(\frac{n_{g,a}L_a}{n_g}\right)^2 \left[\frac{2(1-2\tau_1^*|t_g|t_0 + (\tau_1^* t_0)^2)}{1-(\tau_1^* t_0)^2}\right]^2 \frac{1}{l_r^2}. \tag{S10}$$

In the limit of a weak grating, $|t_g| \to 1$, and near-unity roundtrip transmission, $\tau_1^* t_0 \to 1$, this equation simplifies to

$$\Delta\nu = \Delta\nu_0 \left(\frac{n_{g,a}L_a}{n_g l_r}\right)^2 (1-\tau_1^* t_0)^2. \tag{S11}$$

Note that the finesse of the resonator, $\mathcal{F}$, takes the form [10]:

$$\mathcal{F} = \frac{\pi\sqrt{\tau_1^* t_0}}{1-\tau_1^* t_0} \approx \frac{\pi}{1-\tau_1^* t_0}. \tag{S12}$$

Substituting Eq. S12 and $\eta = \frac{\eta_0}{l_r}$ into Eq. S11 yields

$$\frac{\Delta\nu}{\Delta\nu_0} = \frac{\pi^2}{\mathcal{F}^2}\left(\frac{n_{g,a}L_a}{n_g\eta_0}\eta\right)^2. \tag{S13}$$

Equation S13 represents the linewidth-tuning efficiency trade-off for RE-DBR lasers, which is different from that of DBR lasers (Eq. S9) due to the presence of the resonator finesse $\mathcal{F}$. It is worth noting that our linewidth calculation assumes that the lasing wavelength coincides with the peak reflectivity of the RE-DBR. This assumption simplifies the analysis, as it leads to the cancellation of the first-order dispersion of the RE-DBR reflection. However, it has been shown that the narrowest achievable linewidth in an external cavity laser occurs when there is a finite detuning between the lasing frequency and the resonance [8]. Such detuning introduces dispersion in the RE-DBR reflection, which can compensate for linewidth broadening associated with the amplitude-phase coupling coefficient α_H. As a result, the attainable RE-DBR laser linewidth can be narrower than that predicted by Eq. S13.

Appendix D: Noise characteristics of self-injection-locked and RE-DBR lasers

In the main text, we present experimental demonstration that the RE-DBR laser achieves a narrower linewidth than a self-injection-locked (SIL) laser, despite the latter utilizing a resonator with a quality factor that is an order of magnitude higher. In this section, we provide a theoretical explanation for this counterintuitive result by comparing the theoretical Lorentzian linewidths of both SIL diode lasers and RE-DBR lasers.

1. SIL laser

The Lorentzian linewidth of a laser diode is described by the modified Schawlow-Townes formula [8, 11]:

$$\Delta\nu_{\mathrm{LD}} = \frac{\pi h\nu^3 n_{sp}}{PQ_l^2}(1+\alpha_H^2), \tag{S14}$$

where n_{sp} is the population inversion factor (dimensionless), Q_l is the loaded quality factor of the cold laser cavity, and α_H is the linewidth enhancement factor (dimensionless). Here, we assume that the intrinsic cavity loss is much smaller than the mirror loss, which is typically the case for commercial short-cavity laser diodes. Furthermore, we

consider the scenario where the laser diode has a high-reflectivity facet, and a high-Q resonator is coupled to the opposite high-transmission facet for SIL operation.

The noise reduction factor due to self-injection locking can be expressed as [12]:

$$\frac{\Delta\nu_{\mathrm{SIL}}}{\Delta\nu_{\mathrm{LD}}} = \frac{Q_l^2}{Q_m^2}\frac{1}{16R(1+\alpha_H^2)}, \tag{S15}$$

where $\Delta\nu_{\mathrm{SIL}}$ is the Lorentzian linewidth of the SIL laser, R is the resonator reflectivity, and Q_m is the loaded quality factor of the resonator mode.

By combining Eqs. S14 and S15, the SIL laser linewidth can be written as:

$$\Delta\nu_{\mathrm{SIL}} = \frac{\pi h\nu^3 n_{sp}}{16PQ_m^2}\frac{1}{R}. \tag{S16}$$

2. RE-DBR laser

The noise characteristics of RE-DBR laser have been investigated in Supplementary Section C. Recalling Eqs. S11 and S12, we note that, in the regime of weak grating reflectivity, the Lorentzian linewidth of a RE-DBR laser can be expressed as:

$$\Delta\nu = \Delta\nu_0\left(\frac{n_{g,a}L_a\pi}{n_g l_r\mathcal{F}}\right)^2, \tag{S17}$$

where $\Delta\nu_0$ denotes the linewidth of a FP laser diode with mirror losses matched to those of the RE-DBR, measured at the wavelength corresponding to maximum RE-DBR reflection. The expression for $\Delta\nu_0$ takes the same form as Eq. S14, but with Q_l determined by the peak RE-DBR reflectivity R, yielding:

$$\Delta\nu_0 = \frac{\pi h\nu^3 n_{sp}}{PQ_l^2}(1+\alpha_H^2) = \frac{\pi h\nu^3 n_{sp}}{P}(1+\alpha_H^2)\left[\frac{\lambda\ln R}{4\pi n_{g,a}L_a}\right]^2. \tag{S18}$$

By combining Eqs. S17 and S18, and substituting the resonator finesse with the loaded quality factor of the RE-DBR, $\mathcal{F} = \frac{\lambda Q}{n_g l_r}$, we obtain the Lorentzian linewidth of the RE-DBR laser as:

$$\Delta\nu = \frac{\pi h\nu^3 n_{sp}}{16PQ^2}(1+\alpha_H^2)(\ln R)^2. \tag{S19}$$

Comparing the expressions for the SIL laser linewidth (Eq. S16) and the RE-DBR laser linewidth (Eq. S19), we observe that both linewidths scale inversely with the square of the external cavity quality factor Q (or Q_m). However, their dependence on the feedback strength R differs significantly: the SIL laser linewidth scales as R^{-1}, while the RE-DBR laser exhibits a $(\ln R)^2$ dependence. This distinction is especially pronounced in the weak-feedback regime ($R \ll 1$), where, for identical external cavity Q, the RE-DBR laser achieves a substantially narrower linewidth than the SIL laser. This behavior is illustrated in Fig. S3, where the normalized Lorentzian linewidths of the RE-DBR and SIL lasers are plotted versus the resonator reflectivity. Both plotted linewidths are normalized to $\pi h\nu^3 n_{sp}/16PQ^2$, and the calculations assume a 9-dB m^{-1} waveguide loss and $\alpha_H = 1$. The figure demonstrates that, as the external cavity reflectivity decreases, the RE-DBR laser linewidth increases at a much slower rate than that of the SIL laser, particularly for reflectivities below 4%. These linewidth–reflectivity relationships can be experimentally verified by deliberately increasing the butt-coupling loss—for example, using piezoelectric control to adjust the gap between the gain chip and the external cavity—and measuring the resulting changes in lasing power and linewidth.

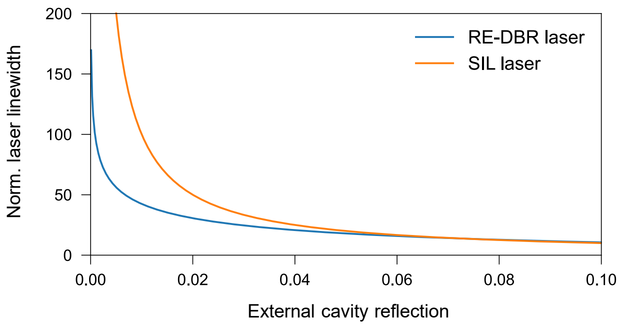

Extended Data Fig. S3: Comparison of the normalized Lorentzian linewidths of the RE-DBR and SIL lasers. Both linewidths are normalized to $\pi h\nu^3 n_{sp}/16PQ^2$, and the linewidth enhancement factor α_H is set to 1. For external cavity reflectivities below 5%, a regime relevant to hybrid integrated lasers with high butt-coupling losses, the RE-DBR laser exhibits a narrower linewidth than the SIL laser.

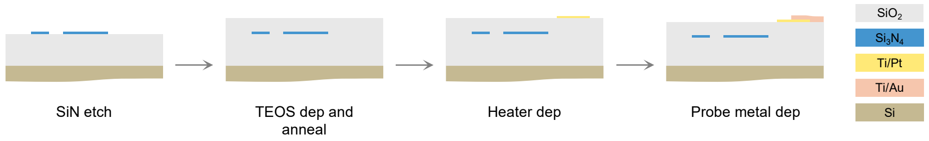

Extended Data Fig. S4: Detailed fabrication process flow of the laser external cavity. The SiN RE-DBR chip is fabricated at the HKUST Nanosystem Fabrication Facility.

Appendix E: Fabrication process of external cavity chip

The fabrication process begins with a commercially available 6-inch-diameter SiN-on-insulator wafer. This wafer consists of three layers: a 100-nm-thick SiN top layer, an 8-um-thick buried oxide layer, and a silicon substrate. The SiN waveguide pattern is defined by an ASML 248-nm deep ultraviolet (DUV) stepper and made permanent using reactive ion etching (RIE). Following this, the wafer is diced into chips, coated with tetraethyl orthosilicate (TEOS) via plasma-enhanced chemical vapor deposition (PECVD), and subjected to annealing at 1,150°C to create a 2-um-thick low-loss oxide cladding. Lastly, the titanium/platinum (Ti/Pt) heaters and titanium/gold (Ti/Au) probe metals are deposited onto the chip using sputtering deposition and patterned with metal liftoff techniques.

Appendix F: Geometry optimization of edge coupler for efficient butt-coupling to RSOA

To achieve high butt-coupling efficiency with the C-band reflective semiconductor optical amplifier (RSOA) from Shenzhen Photonx Technology Co., Ltd., we carefully optimize the geometry of the external cavity's edge coupler. Our edge coupler is a linearly tapered waveguide that bridges the RSOA and the 2.8-μm-wide Si_3N_4 bus waveguide. The optimization process involves two main steps.

First, we focus on optimizing the width of the edge coupler. The RSOA facet has mode field dimensions (MFDs) of 4.2 μm (horizontal) by 2.2 μm (vertical), which are derived from its specification for far-field divergence angle $\theta_{\rm div}$ based on the Gaussian beam model [13]. We calculate the mode overlap between a Gaussian beam with the same MFDs and the mode profile of the tapered waveguide across different taper widths. Our simulations indicate that a taper width of 1200 nm yields a maximum mode overlap of approximately 90%. Next, we optimize the tilt angle of the edge coupler. Applying Snell's law to the interface between the RSOA and the Si_3N_4 chip, $n_{\rm taper}\sin\theta_{\rm taper} = \sin\theta_{\rm RSOA}$, we identify 13° as the optimal tilt angle for the taper. In this equation, $n_{\rm taper} = 1.46$ represents the effective index of the tapered waveguide and $\theta_{\rm RSOA} = 19.5°$ is the lateral beam exit angle for the RSOA.

Appendix G: Simulation of thermo-refractive noise in optical cavity

Thermo-refractive noise (TRN) is a major source of noise in narrow-linewidth semiconductor lasers, usually dominating the laser noise spectrum at offset frequencies ranging from 10 kHz to 100 kHz [14, 15]. In this section, we

describe a numerical simulation approach to model TRN in an optical cavity. Our simulation employs finite element analysis techniques and is based on COMSOL.

1. Fluctuation-dissipation theorem

The calculation of TRN is based on the fluctuation-dissipation theorem. This theorem states that any process that dissipates energy by converting it into heat has a corresponding reverse process linked to thermal fluctuations. For example, imagine a ball falling into water. As the ball slows down due to water friction, its kinetic energy is converted into heat. At the same time, water molecules collide with the ball, causing it to experience random oscillations. These oscillations are examples of thermal fluctuations and illustrate the reverse process of energy dissipation caused by water friction.

Mathematically, the fluctuation-dissipation theorem can be formulated as follows [16, 17]: Consider a system subjected to a generalized force f acting on a generalized coordinate x, and assuming a linear system response, the temporal response is given by:

$$x(t) = \int \alpha(\tau) f(t-\tau) d\tau, \tag{S20}$$

where $\alpha(\tau)$ is the temporal response function. The spectral density of thermal fluctuations in x is given by:

$$S_x(\omega) = 2\hbar \Im[\alpha(\omega)] \coth(\frac{\hbar\omega}{2k_B T}), \tag{S21}$$

where $\Im[\alpha(\omega)]$ is the imaginary part of the Fourier transform of $\alpha(t)$ and corresponds to the system's dissipation rate. This equation indicates that the spectral density of thermal fluctuations $S_x(\omega)$ can be deduced from the corresponding dissipation rate $\Im[\alpha(\omega)]$.

To prepare for the calculation of TRN, we investigate the spectral density of thermal fluctuations in a physical quantity with the specific form: $y = \int x(\mathbf{r}, t) q(\mathbf{r}, t) d^3\mathbf{r}$. According to Levin's formulation [18], when a probe force $f = F_0 \cos(\omega t) q(\mathbf{r})$ is applied to the system, the energy dissipation over a force period $T = 2\pi/\omega$ is proportional to the spectral density of thermal fluctuations of y:

$$S_y(\omega) = 2\hbar \frac{W_{\text{diss}}}{\pi F_0^2} \coth(\frac{\hbar\omega}{2k_B T}). \tag{S22}$$

For this equation to be valid, the probe force f must be energy-conjugate to x. This means that fdx or xdf should represent heat dQ or work dW. For example, temperature T and entropy S form an energy-conjugate pair because $dQ = TdS$. This energy-conjugate relationship is crucial for our subsequent discussion.

2. Thermo-refractive noise in optical cavity

In the context of thermal fluctuations within an optical cavity, the fluctuation-dissipation theorem establishes a relationship between TRN and the rate of optical energy dissipation. This relationship is given by [16, 17]:

$$S_{\delta f/f}(\omega) = 2\hbar \frac{W_{\text{diss}}}{\pi F_0^2} \coth(\frac{\hbar\omega}{2k_B T}), \tag{S23}$$

where $S_{\delta f/f}(\omega)$ is the single-sided spectral density of the normalized fluctuation in the cavity's resonant frequency $\delta f/f$, W_{diss} denotes the energy dissipated over one period $T = 2\pi/\omega$, F_0 is a reference energy set to 1 J, $\hbar$ is the reduced Planck constant, k_B is the Boltzmann constant, and T is the ambient temperature.

To find the dissipated energy W_{diss}, we solve the heat transfer equation where the optical field serves as the heat source:

$$\rho C_V \dot{\delta T} - \kappa \nabla^2(\delta T) = T\dot{S}. \tag{S24}$$

In this equation, ρ is the density, C_V is the specific heat capacity, δT represents the temperature variation, κ is the thermal conductivity, T is the ambient temperature, and S signifies entropy. To convert this differential equation from the time domain to the frequency domain, we substitute $\delta T(t) = \Re(\tilde{T}(\omega)e^{-i\omega t})$ and $S(t) = \Re(\tilde{S}(\omega)e^{-i\omega t})$, yielding:

$$i\omega\rho C_V\tilde{T} + \kappa\nabla^2\tilde{T} = i\omega T\tilde{S}. \tag{S25}$$

Once the amplitude of temperature fluctuation $\tilde{T}$ is determined, the dissipated energy is calculated as:

$$W_{\text{diss}} = \int \frac{\kappa}{T}(\nabla\delta T)^2 d^3\mathbf{r}dt = \int \frac{\pi\kappa}{\omega T}|\nabla\tilde{T}|^2 d^3\mathbf{r}. \tag{S26}$$

To solve Eq. S25, we need to know the expression for $\tilde{S}$. The key is to express the resonant frequency variation δf as a function of δT. Once this relationship is established, the fluctuation-dissipation theorem can be used to derive S, since S is energy-conjugate to the temperature δT.

Using the perturbation theory for the electromagnetic field [19], we have:

$$\frac{\delta f}{f} = -\frac{1}{2}\frac{\int \Delta\epsilon|\mathbf{E}|^2 d^3\mathbf{r}}{\int \epsilon|\mathbf{E}|^2 d^3\mathbf{r}} = -\frac{\int \epsilon_0\sqrt{\epsilon_r}\beta\delta T|\mathbf{E}|^2 d^3\mathbf{r}}{W^{\text{WGM}}}. \tag{S27}$$

Here, ϵ_0 is the permittivity of vacuum, ϵ_r is the relative permittivity, β is the thermo-optic coefficient, W^{WGM} is the normalized factor equal four times the time-averaged electric field energy:

$$W^{\text{WGM}} = \int \epsilon_0\epsilon_r|\mathbf{E}|^2 d^3\mathbf{r}. \tag{S28}$$

Applying the fluctuation-dissipation theorem with $x = \delta T$, $f = S$, $y = \delta f/f$, and $q = -\epsilon_0\sqrt{\epsilon_r}\beta|\mathbf{E}|^2/W^{\text{WGM}}$, the complex amplitude of entropy $\tilde{S}$ is given by:

$$\tilde{S} = -\frac{F_0}{W^{\text{WGM}}}\epsilon_0\sqrt{\epsilon_r}\beta|\mathbf{E}|^2. \tag{S29}$$

In summary, the process of calculating TRN involves the following steps:

1. Start by simulating the distribution of electromagnetic field for the mode of interest, denoted as $\mathbf{E}(\mathbf{r},\omega)$.
2. Use the expression for $\tilde{S}(\mathbf{r},\omega)$ (as given in Eq. S29) to determine the heat dissipation of the electromagnetic field.
3. Solve the heat transfer equation (Eq. S25) to find the temperature variation $\delta T(\mathbf{r},\omega)$.
4. Compute the dissipated energy, W_{diss}, using Eq. S26.
5. Use the fluctuation-dissipation theorem (Eq. S23) to calculate the single-sided spectral density $S_{\delta f/f}(\omega)$.
6. Derive the single-sideband frequency noise spectral density using the formula: $S_{\delta f}(\omega) = f^2 S_{\delta f/f}(\omega)/2$, where f is the eigen-frequency of the optical cavity mode.

3. Simulation of thermo-refractive noise in ring resonator

We conducted a simulation of TRN in a silicon nitride ring resonator using finite element analysis. The resonator consists of a silicon nitride waveguide with a width of 4.6 μm and a thickness of 100 nm. It is surrounded by a 2-μm-thick cladding oxide, with air above and an 8-μm-thick buried oxide below. The ring has a radius of 1 mm, resulting in a resonator free spectral range (FSR) of 30 GHz. The parameters used for the TRN simulation are listed in Table I.

Using these parameters, we simulated TRN in the resonator with COMSOL. The comparison of our simulation result with the experimentally measured laser noise spectrum is present in the text. As is observed in many other

Physical properties	Values
Resonant wavelength of the optical cavity mode	1550 nm
Ambient temperature	298 K
Refractive index of Si_3N_4	1.996
Refractive index of SiO_2	1.444
Thermo-optic coefficient of Si_3N_4	2.45×10^{-5} K^{-1}
Thermo-optic coefficient of SiO_2	8.53×10^{-6} K^{-1}
Specific heat capacity of Si_3N_4	800 J (kg·K)$^{-1}$
Specific heat capacity of SiO_2	705 J (kg·K)$^{-1}$
Specific heat capacity of air	1012 J (kg·K)$^{-1}$
Density of Si_3N_4	3.29×10^3 kg m^{-3}
Density of SiO_2	2.196×10^3 kg m^{-3}
Density of air	1.293 kg m^{-3}
Thermal conductivity of Si_3N_4	30 W (m·K)$^{-1}$
Thermal conductivity of SiO_2	1.38 W (m·K)$^{-1}$
Thermal conductivity of air	0.024 W (m·K)$^{-1}$

TABLE I: Parameter configuration for the simulation of thermo-refractive noise in a SiN ring resonator.

narrow-linewidth lasers, the simulated TRN of the ring resonator matches the laser noise spectrum at offset frequencies between 10 kHz and 100 kHz.

Appendix H: Properties of the resonance mode for self-injection locking

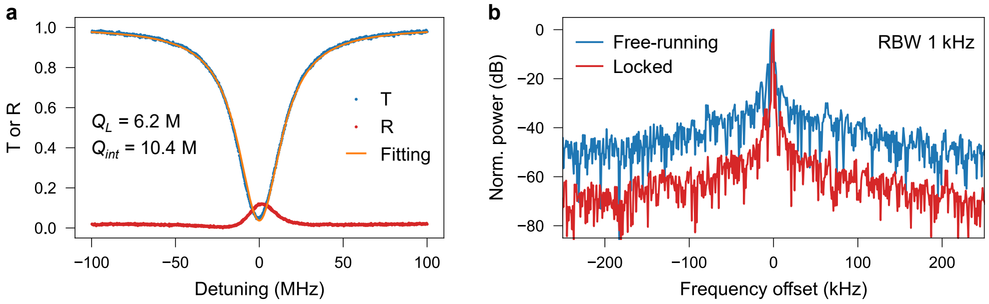

Extended Data Fig. S5: Demonstration of self-injection locking. **a**, Measured transmission and reflection spectra of the resonance mode employed for self-injection locking. The extracted loaded Q is 6.2 million, and the peak reflectivity reaches approximately 10%. **b**, Electrical spectra of the self-heterodyne beatnotes for both free-running and SIL DFB lasers. RBW, resolution bandwidth.

We butt-coupled a distributed feedback (DFB) laser to a SiN ring resonator with a FSR of 30 GHz to realize a self-injection-locked laser. Precise tuning of the laser injection current was employed to align the laser wavelength with a resonant mode of the ring at 1550 nm. The main text presents the noise spectrum of the SIL laser; here, we provide additional characterization of the resonant mode and supporting evidence for SIL. Figure S5a shows the measured transmission and reflection spectra for the relevant resonant mode. The maximum reflection and minimum transmission were determined to be 10% and 5%, respectively. By fitting the experimental data, we extracted a loaded Q of 6.2 million and an intrinsic Q of 10.4 million. Assuming a group index of 1.6 for the 2.8-μm-wide, 100-nm-thick SiN ring, the extracted intrinsic Q corresponds to a waveguide loss of 2.7 dB m^{-1}.

To confirm the occurrence of SIL, we monitored the electrical spectra of delayed self-heterodyne beatnotes while varying the laser injection current. At a specific current setpoint, a pronounced decrease in output power and a marked narrowing of the beatnote linewidth were observed, indicating the onset of SIL. Figure S5b compares the beatnote

spectra in the free-running and locked regimes, where the beatnote linewidth in the locked regime is significantly narrower than that for the free-running regime.

Appendix I: Phase noise spectra of the reference laser for heterodyne detection

In the mode-hop-free tuning test, we employed heterodyne detection to characterize the RE-DBR laser frequency spectrum with high spectral resolution. For this purpose, an external cavity laser diode was used as the reference laser, whose frequency was stabilized by locking it to a fiber Mach-Zehnder interferometer via a proportional–integral–derivative (PID) electronic feedback loop. The single-sideband (SSB) phase noise spectrum of the reference laser was measured using the delayed self-heterodyne method, utilizing a 40-meter-long fiber delay line. The results of these measurements are presented in Fig. S6, which clearly demonstrate that the phase noise in the locked regime is substantially reduced compared to that in the free-running regime.

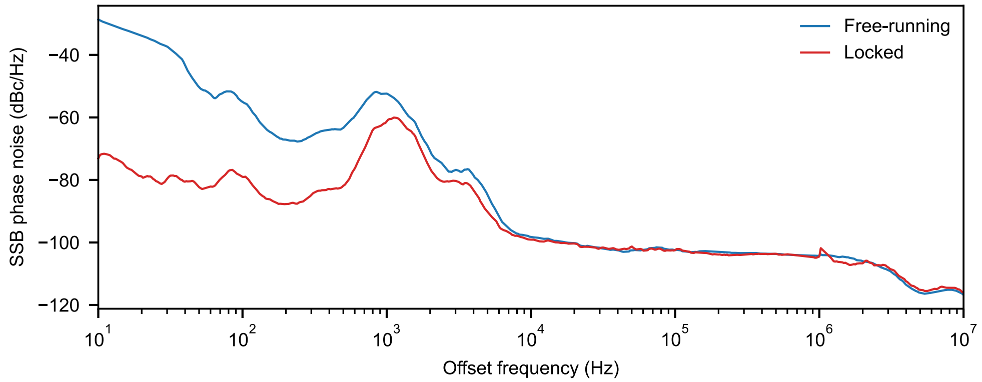

Extended Data Fig. S6: Phase noise spectra of the reference laser. The noise level in the locked regime is significantly lower than that in the free-running regime.

Appendix J: Laser frequency response under direct modulation

The direct modulation characteristics of the RE-DBR laser were investigated. The experimental setup for the direct modulation measurements is depicted in Fig. S7a. In this configuration, the RE-DBR laser was driven by a low-noise current source, superimposed with a sinusoidal modulation signal. The laser output was fiber-coupled and subjected to heterodyne detection, utilizing a frequency-stabilized external cavity laser diode as the reference, as described in Supplementary Section I. The resulting heterodyne beatnote was recorded using an oscilloscope, and its real-time frequency spectrum was extracted via short-time Fourier transform (STFT) analysis.

The dependence of the laser tuning range (i.e., frequency excursion) on the modulation current amplitude was measured for modulation frequencies of 10 kHz, 100 kHz, and 500 kHz, as presented in Fig. S7b. A clear decrease in the achievable tuning range was observed with increasing modulation frequency, reflecting the bandwidth limitations of direct current modulation. At modulation frequencies of 10 kHz and 100 kHz, the tuning range exhibited a linear dependence on the modulation current amplitude, indicating high modulation linearity within this frequency regime. In contrast, at a modulation frequency of 500 kHz, the tuning range reached saturation at a modulation current amplitude of 60 mA, signifying a degradation in modulation linearity at higher modulation frequencies. The corresponding real-time frequency spectra at 10 kHz, 100 kHz, and 500 kHz, shown in Fig. S7c, further illustrate the frequency-dependent behavior of the modulation linearity.

There is considerable room for improvement in frequency excursion, modulation bandwidth, and linearity. For instance, the frequency excursion demonstrated here is limited because current modulation induces mismatches between the laser's longitudinal mode and the external cavity resonance. This limitation may be overcome by applying modulation directly to the external cavity rather than to the injection current. Additionally, both modulation bandwidth and linearity are constrained by the intrinsic properties of current modulation. These characteristics could be significantly enhanced by implementing external electro-optic modulation, such as through a lithium niobate-based RE-DBR structure.

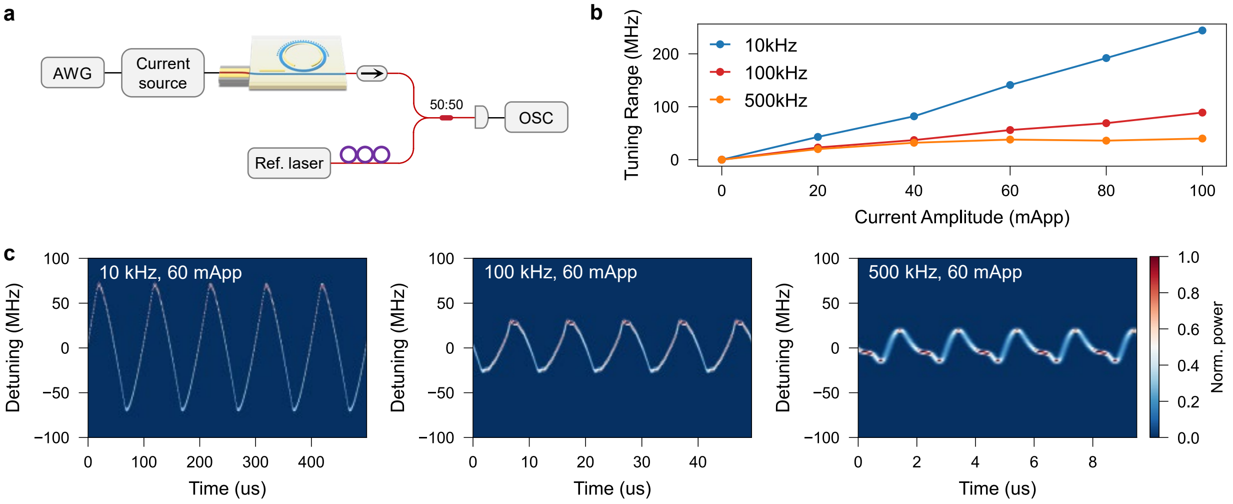

Extended Data Fig. S7: Current modulation performance of the RE-DBR laser. **a**, Schematic of the experimental setup for current modulation measurements. The RE-DBR laser is driven by a low-noise current source with sine wave modulation, and heterodyne detection is employed. The resulting beatnote is analyzed using a short-time Fourier transform to extract its frequency spectrum. **b**, Measured laser frequency tuning range as a function of modulation frequency and current amplitude. **c**, Real-time frequency response of the laser at a current amplitude of 60 mA for modulation frequencies of 10 kHz (left), 100 kHz (center), and 500 kHz (right). AWG, arbitrary waveform generator; OSC, oscilloscope.

Appendix K: Thermal stability of RE-DBR

The reflection spectra of the RE-DBR measured over a temperature range from 20 °C to 50 °C are shown in Fig. S8. As the temperature increases, the reflection peak exhibits a linear wavelength shift, corresponding to a total thermo-optic tuning range of 0.4 nm for a 30 °C change. Notably, the fluctuations in peak reflectivity remain below 0.5 dB throughout the entire temperature range, indicating that the feedback strength of the RE-DBR is largely insensitive to temperature variations. This behavior is consistent with theoretical expectations, as both the resonance mode and the Bragg wavelength are anticipated to shift at identical rates with temperature, thereby preserving their spectral overlap and resulting in a stable peak reflectivity. The demonstrated thermal stability of the RE-DBR is crucial for achieving high laser power stability during thermo-optic tuning, as discussed in the main text.

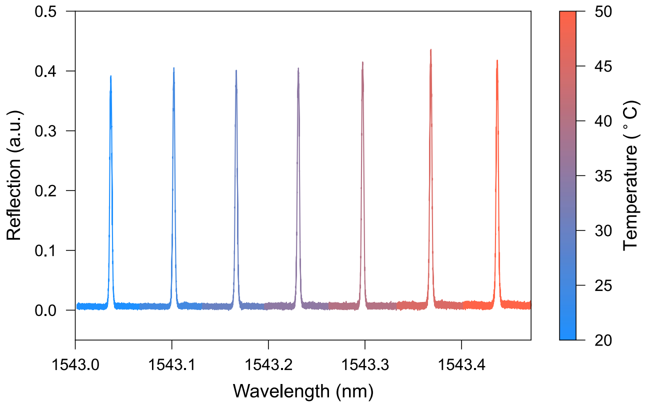

Extended Data Fig. S8: Reflection spectra of the RE-DBR measured at various temperatures. The peak reflectivity exhibits fluctuations of less than 0.5 dB over a 30 °C temperature range, indicating excellent thermal stability.

[1] Kang, Y. M., Arbabi, A. & Goddard, L. L. A microring resonator with an integrated bragg grating: a compact replacement for a sampled grating distributed bragg reflector. *Optical and quantum electronics* **41**, 689–697 (2009).
[2] Kang, Y. M., Arbabi, A. & Goddard, L. L. Engineering the spectral reflectance of microring resonators with integrated reflective elements. *Optics Express* **18**, 16813–16825 (2010).
[3] Okamoto, K. *Fundamentals of optical waveguides* (Elsevier, 2021).
[4] Jin, W. *Ultra-low-loss silicon nitride photonic integrated circuits for highly coherent lasers* (University of California, Santa Barbara, 2022).
[5] Wenzel, H., Guther, R., Shams-Zadeh-Amiri, A. & Bienstman, P. A comparative study of higher order Bragg gratings: coupled-mode theory versus mode expansion modeling. *IEEE Journal of Quantum Electronics* **42**, 64–70 (2005).
[6] Yu, D. Semi-analytical model for resonator-enhanced distributed bragg reflector. `https://nagato-d.github.io/notes_optoelectronics_advanced/note-redbr/` (2025).
[7] Kazarinov, R. & Henry, C. The relation of line narrowing and chirp reduction resulting from the coupling of a semiconductor laser to passive resonator. *IEEE Journal of quantum electronics* **23**, 1401–1409 (2003).
[8] Tran, M. A., Huang, D. & Bowers, J. E. Tutorial on narrow linewidth tunable semiconductor lasers using Si/III-V heterogeneous integration. *APL Photonics* **4** (2019).
[9] Jin, W., Polcawich, R. G., Morton, P. A. & Bowers, J. E. Piezoelectrically tuned silicon nitride ring resonator. *Optics Express* **26**, 3174–3187 (2018).
[10] Bogaerts, W. *et al.* Silicon microring resonators. *Laser & Photonics Reviews* **6**, 47–73 (2012).
[11] Schawlow, A. L. & Townes, C. H. Infrared and optical masers. *Physical review* **112**, 1940 (1958).
[12] Kondratiev, N. *et al.* Self-injection locking of a laser diode to a high-Q WGM microresonator. *Optics Express* **25**, 28167–28178 (2017).
[13] Rayleigh length — Wikipedia, the free encyclopedia. `https://en.wikipedia.org/wiki/Rayleigh_length`.
[14] Jin, W. *et al.* Hertz-linewidth semiconductor lasers using CMOS-ready ultra-high-Q microresonators. *Nature Photonics* **15**, 346–353 (2021).
[15] Xiang, C. *et al.* 3D integration enables ultralow-noise isolator-free lasers in silicon photonics. *Nature* **620**, 78–85 (2023).
[16] Huang, G. *et al.* Thermorefractive noise in silicon-nitride microresonators. *Physical Review A* **99**, 061801 (2019).
[17] Kondratiev, N. & Gorodetsky, M. Thermorefractive noise in whispering gallery mode microresonators: Analytical results and numerical simulation. *Physics Letters A* **382**, 2265–2268 (2018).
[18] Levin, Y. Internal thermal noise in the LIGO test masses: A direct approach. *Physical Review D* **57**, 659 (1998).
[19] Meade, R. D. V., Johnson, S. G. & Winn, J. N. Photonic crystals: Molding the flow of light (2008).
